# Bubbling With Confidence: A Study of Numerous Deep Anterior Lamellar Keratoplasty Cases Using Anwar’s Big Bubble Technique

**DOI:** 10.7759/cureus.46528

**Published:** 2023-10-05

**Authors:** Abdul Basit, Hamid Nafees, Bakht D Khan, Mir Z Marwat, Sofia Iqbal, Saud A Rehman, Muhammad Abdullah

**Affiliations:** 1 Ophthalmology, Hayatabad Medical Complex Peshawar, Peshawar, PAK; 2 Ophthalmology, University Hospital Galway, Galway, IRL; 3 Ophthalmology, Pak International Medical College, Peshawar, PAK; 4 Ophthalmology, Jinnah Medical College Peshawar, Peshawar, PAK; 5 Ophthalmology, Jinnah Medical and Dental College, Peshawar, PAK

**Keywords:** kerato-refractometry, therapeutic penetrating keratoplasty, keratoconus (kc), big bubble, deep anterior lamellar keratoplasty

## Abstract

Purpose: This study aims to assess the postoperative results, variability, and complications of a hundred deep anterior lamellar keratoplasty cases.

Study design and duration: This is an observational study. The study was conducted at Pak International Medical College (PIMC) for a duration of four years (January 2019-January 2023).

Methodology: Our study collected information on a hundred cases of deep anterior lamellar keratoplasty (DALK) utilizing Anwar's big bubble technique, consisting of patients with keratoconus, superficial corneal scars, and macular dystrophy. Consenting patients had their pre and postoperative visual acuities and keratometry readings recorded. Overall success and complications were recorded and compared with the present literature.

Results: Big bubble formation was achieved in 87% (n = 87) eyes and not achieved in 13% (n=13). There was a significant reduction in keratometry values after the procedures as well as improved vision in all patients, with 84% reporting significant improvement. Descemet membrane exposure was achieved in 91% (n=91). Complications included the failure of Anwar’s big bubble formation in 13% (n=13) patients and the failure to expose Descemet’s in nine patients (9%).

Conclusion: DALK using the big bubble technique is a safe and effective procedure in patients with corneal diseases who have a healthy Descemet membrane and endothelium.

## Introduction

Pathologies affecting corneal architecture are impartial when it comes to ethnicities, genders, and parts of the world where they are prevalent. Keratoconus is especially notorious for its distortion of the cornea, which does not improve with spectacles and is irreversible [1]. While it may be possible to thwart progression, the options get limited to keratoplasty and its types once it leads to thinning or scarring [2].

Deep anterior lamellar keratoplasty (DALK) has numerous advantages over penetrating keratoplasty (PK) when it comes to postoperative best-corrected vision [3]. Others include greater wound stability/strength postoperatively, a lower chance of graft rejection, less astigmatism, shorter use of steroids, fewer chances of intraocular infection, early removal of sutures, and early rehabilitation [4]. Since it is a closed-globe procedure, there is less chance of grave intraocular complications, such as intraoperative expulsive hemorrhage and uveal/lens prolapse. DALK is a surgical technique that involves the removal of the anterior segment of the cornea, leaving the posterior lamella intact; the only real drawback compared to PK is the steeper learning curve [5].

Anwar’s big bubble technique utilizes the creation of a big bubble in the stroma using air injection. This technique allows for smoother and easier separation of the corneal layers, thus facilitating the removal of the pathological cornea [6]. The use of Anwar’s big bubble technique has been reported to have several advantages compared to other techniques of deep anterior lamellar keratoplasty, including a quicker operating time, a reduced risk of perforation, and improved visual outcomes [7-8].

The purpose of this study is to evaluate the outcomes of DALK using Anwar’s big bubble technique in a cohort of 100 patients with various corneal disorders in which healthy Descemet and endothelium were present. We aim to assess the surgical outcomes, including visual acuity and corneal astigmatism, as well as the intraoperative and postoperative complications associated with Anwar’s big bubble technique.

This study may add valuable information on the safety, efficacy, and reproducibility of Anwar’s big bubble technique in the management of corneal disorders. Furthermore, this study may serve as a reference for ophthalmologists who wish to adopt this technique in their clinical practice.

## Materials and methods

This study was carried out at Pak International Medical College (PIMC), Peshawar, Pakistan, from January 2019 to January 2023 on patients who were admitted fulfilling the criteria for DALK. 90% (n=103) had grade 4 keratoconus (using the Amsler-Krumeich classification), while 7% (n=08) had superficial corneal scarring or opacity with sparing of the deeper corneal layers, and 3% (n=3) had macular corneal dystrophy. Only a hundred eyes were included in the final results. Every patient underwent corneal topography, keratometry, slit lamp biomicroscopy, best corrected visual acuity (BCVA), detailed ocular examination, and other baseline investigations. Donor corneas with healthy epithelium and stroma were arranged with the availability of a backup cornea in all cases as well. Preoperative keratometry values and presenting visual acuity were recorded. 60% (n=60 with males n=37 and females n=23) had profound visual impairment (BCVA logmar <1.3 and Snellen <3/60), and 40% (n=40 with males n=29 and females n=11) had severe visual impairment (BCVA logmar <1-1.3 and Snellen <6/60-3/60). Those patients whose transplant failed or was converted to penetrating keratoplasty were not included. The study was performed in accordance with the Declaration of Helsinki. On August 5, 2019, the Pak International Medical College's Ethical Review Board approved the study with ref no. 1/19/DMR/PIMC. After being explained the purpose and method of the study, patients who gave oral and written consent were included in the study.

Surgical procedure

**Figure 1:**
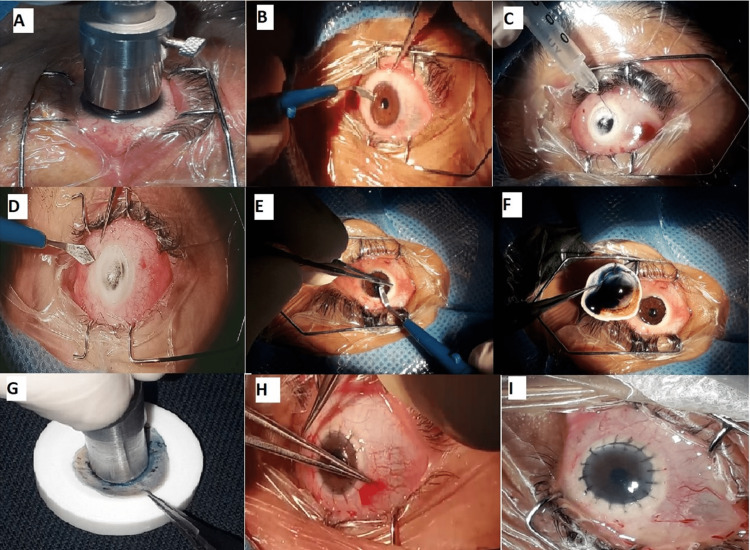
Steps of deep anterior lamellar keratoplasty procedure using Anwar's big bubble technique

The patient is given peribulbar local anesthesia with a combination of xylocaine and adrenaline. General anesthesia was given in nine cases, all in the 10-15 age group (Table 1). The surgeon marks the size of the trephination on the cornea and then uses the required size of the trephine to create a circular incision on the cornea (Figure 1A). The surgeon performs a controlled partial thickness trephination of the superficial cornea as deep as possible using a simple corneal trephine (Figure 1B). The surgeon creates a big bubble by injecting air or gas with a 27-gauge needle into the deep corneal layers to separate Descemet's membrane and endothelium from the overlying stroma (Figure 1C). Using a crescent knife, the surgeon deepens the superficial wound to expose the deeper corneal layers, avoiding damage to Descemet's membrane (Figure 1D).

**Table 1:** Age and gender distribution n = number of patients, % = percentage of patients of total

S. no.	Age in years	No. of patients	% age	Male/female (n)
1	10–15 years	21	(21%)	15/6
2	15–20 years	31	(31%)	21/10
3	20–25 years	33	(33%)	24/9
4	Above 25 years	18	(15%)	8/7
5	Total	100	(100%)	68/32

A small incision is made at the limbus to release any residual fluid and reduce the intraocular pressure (Figure 1E). The surgeon removes the stromal tissue step by step using a crescent knife, aiming to preserve Descemet's membrane (Figure 1E-1F). Once the stromal tissue has been removed, Descemet's membrane is exposed, appearing clear and shiny (Figure 1F). The surgeon prepares the donor cornea by removing the Descemet's membrane and endothelium using a technique that stains the endothelium with sipic blue and then peeling the Descemet-endothelium complex with a plain or tooth forceps (Figure 1G). The surgeon uses a trephine of the same size as the recipient's cornea to create a circular incision in the donor's cornea. The surgeon sutures the donor cornea onto the recipient bed using interrupted 10/0 nylon sutures (Figure 1H-1I).

After the procedure, the patient is closely monitored for any complications, such as intraocular pressure elevation or infection. The patient is put on antibiotics and steroid eye drops, as well as oral antibiotics, depending on the case requirements, and follow-up appointment dates with the surgeon are assigned to monitor the progress of the recovery. The patients were followed up on the first post-op day, then weekly for two weeks, then monthly for six months in cases of uneventful recovery.

## Results

Hundred and fourteen patients (eyes) whose preoperative measurements were taken initially underwent the DALK procedure in our study. Only a hundred cases were included in the results, and the following text talks specifically about the hundred cases. Out of these, 68% (n=68) were male patients, and 32% (n=32) were female. The mean average age of the patients was 19.86±6.93, with the stratified ages given in Table 1. Out of these, 90% (n=90) suffered from high-grade keratoconus, 7% (n = 7) had superficial corneal scarring, and 3% (n=3) had macular corneal dystrophy.

The Anwar’s big bubble technique we utilized resulted in successful big bubbles in 87% (n=87) eyes and failed in 13% (n=13) eyes. However, a bare, shiny Descemet membrane was achieved in 91% (n=91) of the eyes, with 9% (n=09) resulting in pre-Descemetic exposure.

The mean preoperative K1 (flat) value was 56.3±8.8D, and the mean postoperative K1 value after DALK was 43.56±2.60D. The mean preoperative K2 (steep K reading) value is 63.6±8.5D, and the mean postoperative K2 value after DALK is 45±2.33D. These data are very compelling, keeping in mind the goal of DALK surgery, whose aim is to reduce corneal curvature in certain disorders (Table 2).

**Table 2:** Pre and postoperative keratometry readings SD: standard deviation

Keratometric reading	Sample mean with standard deviation
K1 (flat K reading) preoperative	56.3±8.8D
K2 (steep K reading) preoperative	63.6±8.5D
K1 (flat) postoperative	43.56±2.60D
K2 (steep) postoperative	45±2.33D

The presenting visual acuities were 60% (n=60 with male n=39 and female n=21) had profound visual impairment (BCVA logmar <1.3 and Snellen <3/60) and 40% (n=40 with male n=29 and female n=11) had severe visual impairment (BCVA logmar <1 to 1.3 and Snellen <6/60 to >3/60). Postoperative results were gratifying with 84% (n=84) having normal (6/36 or better) visual acuity and 16% (n=16) having vision impairment (<6/36) on follow-up. They are summarized in Table 3.

**Table 3:** Pre- and postoperative visual impairment in patients n = number of patients, % = percentage of patients (n=%)

Visual acuity	Percentage		
	Male (n,%)	Female (n,%)	Total
Profound visual impairment (preoperative)	39	21	60%
Severe visual impairment (preoperative)	29	11	40%
Vision impairment (postoperative)	11	5	16%
Normal (postoperative)	57	27	84%

Intra and postoperative complications related to the procedure included failure for the big bubble to form in 13 eyes (n=13/114) and failure to expose the Descemet’s membrane in 9 (n=09/114) eyes. Microperforation was noted in 10 (n=10/100) patients, while macroperforation was in 14 eyes; these 14 were excluded out of the final hundred used in the analysis. Microperforations were holes that did not collapse the anterior chamber, while macroperforations were punctures after which the anterior chamber could not be maintained with air or salt solutions. Macroperforations were converted into penetrating keratoplasty and thus excluded as cases as far as this study was concerned. Suture-related complications were in 10 patients (n=10/100 after the 14 patients converted to PK were ruled out), and the persistent epithelial defect was in 14 patients (n=14/100). An interface haze was found in eight patients (n=8/100), while one had the formation of a double anterior chamber. Topical dexamethasone 0.1% was used for clearance of interface haze, while the double anterior chamber resolved spontaneously. The complications are summarized in Table 4.

**Table 4:** Complications encountered by patients n = number of patients in which the complication occurred, N = total eyes before or after excluding conversions to PK, % = percentage of total

S.no	Complications	No. of eyes(n)/total patients (N)	Percentage (%)
1	Failure of Anwar's big bubble formation	13/114	11.4%
2	Failure to expose Descemet’s membrane	9/114	7.9%
3	Macroperforation	14/114	12.2%
4	Mircoperforation	10/100	10%
5	Suture-related complications	10/100	10%
6	Suture-related endophthalmitis	2/100	2%
7	Epithelial defect	14/100	14%
8	Interface haze	8/100	8%
9	Double anterior chamber	1/100	1%

## Discussion

Literature's first record of lamellar keratoplasty dates back to the 19th century, with the first attempts to perform the procedure by dissecting Descemet’s membrane coming in 1959 [9-10]. With Anwar's discovery that injecting stromal air causes localized separation of the Descemet’s membrane in 2002, a new technique with a less steep learning curve and better postoperative outcomes was introduced to ophthalmologists worldwide [6]. Even though it is worth noting that injecting air at different steps was attempted several times in the past, Anwar’s technique had good outcomes that were easily reproducible. Our study utilizes this technique and implicates its advantages in a developing country like ours, where resources are limited.

Our preoperative keratometry levels were K1=56.3±8.8D and K2=63.6±8.5D, while the postoperative keratometry levels were K1=43.56±2.60D and K2=45±2.33D. This significant decrease in corneal steepness is beneficial for patients and compares to statistics available in the literature. In Anwar’s original study, he reported a mean preoperative K2 value of 59.1D and a mean postoperative K2 value of 45.7D in a cohort of 203 eyes that underwent DALK with the big bubble technique [6]. Noble et al. studied the visual outcomes and complications of deep anterior lamellar keratoplasty in a cohort of 191 eyes. This study reported a mean preoperative K2 value of 56.9D and a mean postoperative K2 value of 45.4D in a cohort of 191 eyes that underwent DALK [11].

In studies comparing DALK to PK, the DALK outcomes were statistically better. The meta-analysis by Zhang et al. in 2015 combined 16 studies comparing outcomes. The study reported a mean preoperative K2 value of 56.1 D and a mean postoperative K2 value of 45.5 D for the DALK group, a mean preoperative K2 value of 56.5 D, and a mean postoperative K2 value of 44.5 D for the PK group [12]. The advantages of DALK are well documented in the literature [13-15]. A study by Hakeem in 30 eyes comparing manual versus big bubble DALK found better postoperative BCVA in the big bubble variety [16].

In our study, we achieved Anwar’s big bubble formation in 87% of the eyes, which was very favorable and comparable to that achieved by Anwar and Teichmann in their original study (85%) [6]. A trend can be noticed in studies done over the years in which the rate of successful bubble formation is improving. Fogla and Padmanabhan reported 60% in their interventional case series of 13 eyes in 2006 [17]. Han et al. reported 76.4% success in 110 DALK procedures, while Sepher's study showed a success rate of 79.2% in 289 eyes [18-19]. More recent studies have shown better rates, i.e., Fogla's repeat study with a modified technique showed a success rate of 95% [20]. There have been factors implicated in achieving successful bubble formation, including surgeon skill, recipient sex, and trephination size [21]. Other well-documented factors include the type of technique used for DALK, corneal thickness and curvature, the degree and location of the cone in keratoconus patients, and corneal scarring or opacities [19,22].

Our complications in this study are given in Table 4 of the results section; they were managed as follows. Failure of big bubble formation leads to manual exposure of the Descemets membrane. Failure to expose the bare Descemets membrane resulted in pre-Descemetic DALK, and macroperforations were converted to penetrating keratoplasty and excluded from the study. Microperforations proceeded as long as the anterior chamber was formed. Suture-related complications were treated with antibiotics, while interface haze was treated with dexamethasone eye drops. There was one case of a double anterior chamber, which resolved spontaneously over a period of two weeks.

The limitations of our study include that it is an observational study conducted in a single center, which might cause a selection bias in the patients who are present at the center. A randomized control trial with a control group helps establish more concrete causal relationships. Surgeon skill for the DALK procedure as well as external factors like postoperative care, compliance, and prevalent co-morbidities could have introduced variability in the study. All the procedures were performed by the same surgeon for our study, and negative results (conversions to penetrating keratoplasty) were also mentioned to eliminate any operator or publication bias, respectively.

## Conclusions

Our study aimed to shed some light on the outcomes, variability, and complications of the DALK procedure. Keratoplasty is a sight-saving, life-changing intervention, and donor corneas are an invaluable resource, so the best possible techniques should be developed and utilized. DALK using the big bubble technique is a safe and effective procedure for patients with corneal diseases who have healthy Descemet’s membrane and endothelium. The amount of pre- and postoperative complications compared to penetrating keratoplasty gives it a clear edge where possible, and because of better-improved postoperative BCVA, it is superior to both penetrating keratoplasty and the manual techniques of DALK. A longer follow-up with these patients could further help unmask long-term outcomes and complications.
